# Overall performance of a drug–drug interaction clinical decision support system: quantitative evaluation and end-user survey

**DOI:** 10.1186/s12911-022-01783-z

**Published:** 2022-02-22

**Authors:** Greet Van De Sijpe, Charlotte Quintens, Karolien Walgraeve, Eva Van Laer, Jens Penny, Greet De Vlieger, Rik Schrijvers, Paul De Munter, Veerle Foulon, Minne Casteels, Lorenz Van der Linden, Isabel Spriet

**Affiliations:** 1 Pharmacy Department, University Hospitals Leuven, Leuven, Belgium; 2 Clinical Pharmacology and Pharmacotherapy, Department of Pharmaceutical and Pharmacological Sciences, KU Leuven, Leuven, Belgium; 3 Department of Information Technology, University Hospitals Leuven, Leuven, Belgium; 4 Department of Intensive Care Medicine, University Hospitals Leuven, Leuven, Belgium; 5 Department of General Internal Medicine, University Hospitals Leuven, Leuven, Belgium; 6 Department of Microbiology, Immunology and Transplantation, KU Leuven, Leuven, Belgium

**Keywords:** Drug interactions, Drug–drug interaction, Clinical decision support systems, Alert fatigue

## Abstract

**Background:**

Clinical decision support systems are implemented in many hospitals to prevent medication errors and associated harm. They are however associated with a high burden of false positive alerts and alert fatigue. The aim of this study was to evaluate a drug–drug interaction (DDI) clinical decision support system in terms of its performance, uptake and user satisfaction and to identify barriers and opportunities for improvement.

**Methods:**

A quantitative evaluation and end-user survey were performed in a large teaching hospital. First, very severe DDI alerts generated between 2019 and 2021 were evaluated retrospectively. Data collection comprised alert burden, override rates, the number of alert overrides reviewed by pharmacists and the resulting pharmacist recommendations as well as their acceptance rate. Second, an e-survey was carried out among prescribers to assess satisfaction, usefulness and relevance of DDI alerts as well as reasons for overriding.

**Results:**

A total of 38,409 very severe DDI alerts were generated, of which 88.2% were overridden by the prescriber. In 3.2% of reviewed overrides, a recommendation by the pharmacist was provided, of which 79.2% was accepted. False positive alerts were caused by a too broad screening interval and lack of incorporation of patient-specific characteristics, such as QTc values. Co-prescribing of a non-vitamin K oral anticoagulant and a low molecular weight heparin accounted for 49.8% of alerts, of which 92.2% were overridden. In 88 (1.1%) of these overridden alerts, concurrent therapy was still present. Despite the high override rate, the e-survey revealed that the DDI clinical decision support system was found useful by prescribers.

**Conclusions:**

Identified barriers were the lack of DDI-specific screening intervals and inclusion of patient-specific characteristics, both leading to a high number of false positive alerts and risk for alert fatigue. Despite these barriers, the added value of the DDI clinical decision support system was recognized by prescribers. Hence, integration of DDI-specific screening intervals and patient-specific characteristics is warranted to improve the performance of the DDI software.

**Supplementary Information:**

The online version contains supplementary material available at 10.1186/s12911-022-01783-z.

## Background

Medication errors are an important threat to patient safety. They can result in adverse drug events, which occur in 6 to 19% of hospitalized patients and which might negatively impact the patient’s health status as well as increase overall costs [1–4]. Up to one-half of adverse drug events are due to medication errors and are thus preventable [1, 4, 5]. More specifically, 5–17% of adverse drug events among inpatients are caused by drug–drug interactions (DDIs) [1, 6].

Substantial efforts have already gone into reducing medication errors, including providing software support to prescribers [7]. Yet, medication errors remain relatively common, particularly during prescribing, accounting for 7% of all prescriptions in inpatients [8].

Computerized physician order entry (CPOE) linked with clinical decision support systems (CDSSs) has seen a broad uptake in many hospitals to prevent medication errors and associated harm [9]. CDSSs analyze structured data available in the CPOE and provide guidance for drug related problems including DDIs, maximum doses, duplicate therapy and drug allergies. CPOE/CDSS can approximately halve the risk of medication errors and potential adverse drug events [10–12].

Despite these benefits, CDSSs are often basic and associated with a high burden of false positive alerts and alert fatigue. Alert fatigue might cause prescribers to override both irrelevant and clinically relevant alerts due to an overload of irrelevant alerts, which compromises the desired safety effect of the CDSSs [13]. Alert override rates ranging from 49 to 96% have been described [13]. To reduce alert fatigue, the specificity of the alerts should be improved. This can be accomplished by including more patient-specific and context-specific information into clinical decision support algorithms, which upgrades the basic CDSS to a more advanced level. Different studies have shown a substantial reduction in DDI alert burden by applying patient and context-specific factors [14–18]. Moreover, to further improve the system, prescribers’ perspectives on overall CDSS usefulness and reasons behind the poor alert uptake should be evaluated as well [19].

In Belgium, the implementation of a DDI CDSS is promoted by the Belgian Meaningful Use Criteria (BMUC). BMUC was set up by the Belgian government in 2016 to accelerate the adoption of integrated electronic health records by providing financial incentives. In the University Hospitals Leuven (UZ Leuven), basic CDSSs including a DDI screening and alerting module have been implemented in the CPOE as of 2009. However, it remains of utmost importance that hospitals make critical decisions concerning the implementation of a basic DDI CDSS to prevent alert fatigue [20–22].

The aim of this study is to perform a quantitative analysis of the current DDI CDSS as well as an end-user survey study, in order to identify barriers and opportunities for improvement of this system.

## Methods

### Study design

This study comprises a quantitative evaluation and end-user survey. First, a retrospective study was carried out on data generated over a 2-year period (January 2019 to January 2021). All medication orders prescribed in the CPOE for non-critically ill hospitalized patients and patients admitted to the day care hospital during the study period were included in this analysis. Second, a cross-sectional survey study was carried out in February 2021. The study was approved by the Ethics Committee Research UZ/KU Leuven (S63862).

### Setting

The study took place using data from UZ Leuven, a tertiary 1995-bed teaching hospital in Belgium. The home-grown hospital information system with electronic patient records for non-critically ill patients (Klinisch Werkstation (KWS)®, Nexuzhealth) integrates both CPOE and CDSSs. KWS is currently used in 38 other Belgian hospitals or healthcare institutions.

#### CDSS for DDI

In the CPOE, a specific CDSS screening and alerting module for DDIs was implemented in April 2009. The knowledge base used for this module is the commercially available DelphiCare® database (Belgian Pharmacists Association, Belgium), which is based on the ABDATA database (ABDATA Pharma-Daten-Service, Germany). In the DelphiCare® database, DDIs are divided into 8 categories based on the clinical severity and relevance of the interaction, with a lower value signifying a higher DDI severity. In UZ Leuven, DDIs with a severity of category 1, 2 or 3 according to the DelphiCare® database are reviewed and evaluated by an expert working group consisting of physicians and pharmacists. These DDIs are then re-categorized in three risk groups: “very severe”, “severe” and “other” DDIs taking into account the available evidence and the severity of potential consequences of the DDI. The results of in-house re-categorization are shown in Additional File 1. Very severe DDIs generate an interruptive alert during prescribing, requiring a free-text override motivation with password confirmation to continue the medication order. Severe DDIs generate an interruptive pop-up alert, but do not need to be overridden. Alerts for other DDIs are not interruptive but can be consulted by the prescriber via an informative ribbon bar in the CPOE. All DDIs with a severity of 4–8 according to the DelphiCare® database are automatically categorized in the “other” group.

A DDI alert contains information on its severity level, involved drugs, potential clinical consequences, and the option to receive detailed information regarding the DDI, including pharmacological effect, mechanism, management options, risk factors and literature references. In order to receive this detailed information, actively pushing a button “more information” is needed.

A fixed screening interval of 7 days before and after prescribing a drug in the CPOE is used to screen for DDIs. When two interacting drugs are prescribed within this screening period of 14 days, even when there is no actual co-prescription, an alert will be generated.

#### Check of medication appropriateness service

In UZ Leuven, a pharmacy-based service called "Check of Medication Appropriateness" (CMA), has been implemented in adjunct to the existing CDSS modules to further improve medication surveillance and provide pharmacotherapeutic support when indicated [23]. The CMA comprises a clinical rule-based screening for potentially inappropriate prescriptions, followed by a medication review performed by trained clinical pharmacists (i.e. “CMA pharmacists”). If deemed necessary, recommendations are provided directly in the electronic patient record addressing the treating physician. In case of potentially severe risk, the physician is also contacted by phone. The CMA is performed on a daily basis (0.5 full-time equivalents), except on Sunday.

One of the rules of the CMA relies on the secondary review of alert overrides for very severe DDIs. The override motivations provided by prescribers are assessed by the CMA pharmacist. Recommendations to the prescriber are then provided when the DDI is considered to be clinically relevant. After an overridden DDI alert for a specific patient is reviewed by the pharmacist, it is muted for 14 days in the CMA service.

**Fig. 1:**
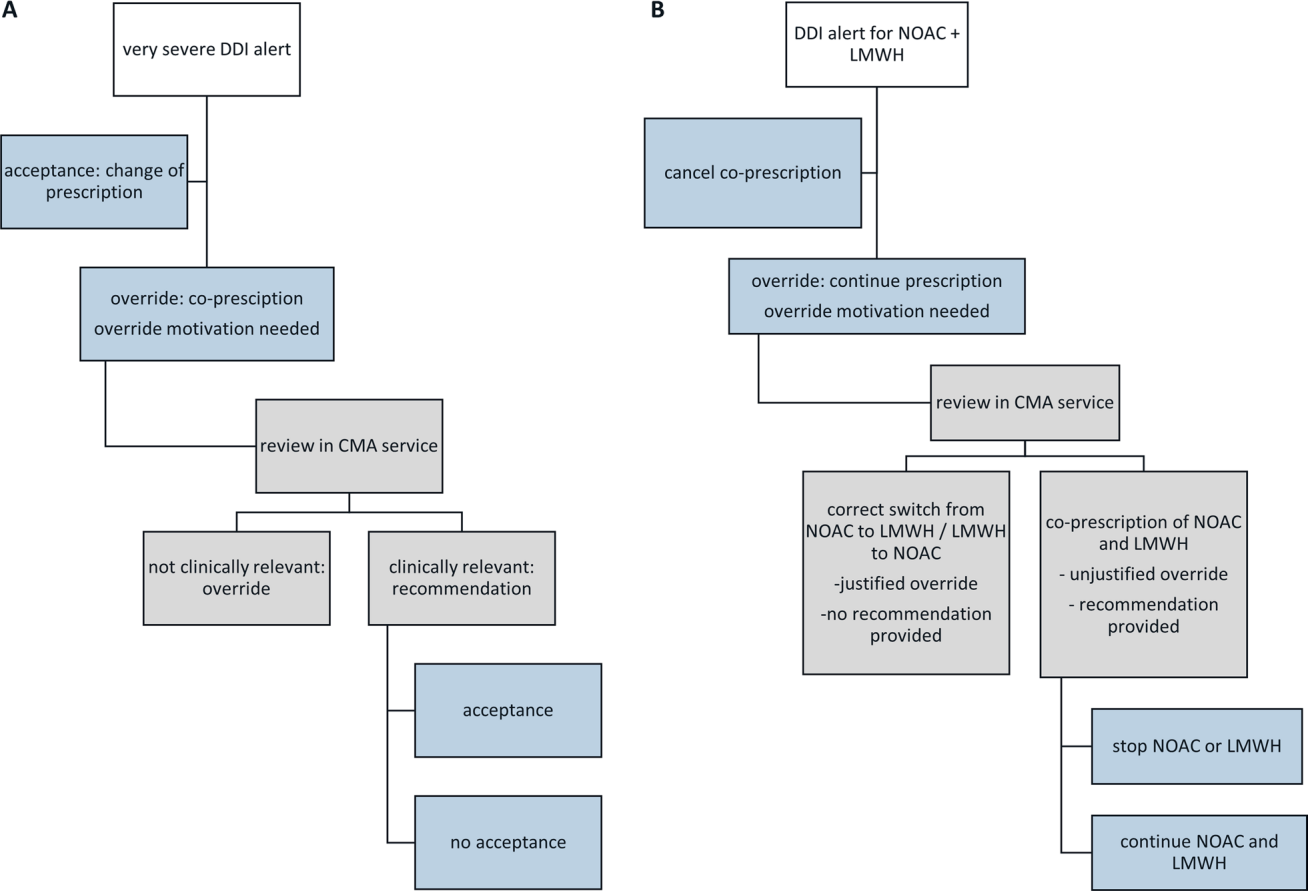
Actions that must be completed by prescribers and pharmacists when an alert for a very severe drug–drug interaction is generated. Panel **A**: general workflow diagram of very severe drug–drug interaction alerts. Panel **B**: workflow diagram using a specific example categorized as a very severe drug–drug interaction: co-prescription of a non-vitamin K oral anticoagulant and a low molecular weight heparin. Blue: actions that must be taken by the prescriber. Grey: actions that must be taken by the pharmacist. DDI, drug–drug interaction, NOAC, non-vitamin K oral anticoagulant; LMWH, low molecular weight heparin

Figure 1 shows the complete process of actions that must be completed by prescribers and pharmacists when an alert for a very severe DDI is generated.

### Data collection

#### Quantitative evaluation

Data were extracted from the electronic patient record. A log file of very severe DDI alerts was collected. Alert burden, initial acceptance and override rates were determined in total and for each DDI pair.

A log file of overridden very severe DDIs reviewed by the CMA pharmacists was collected. The number of alert override reviews, the number of pharmacist recommendations and their acceptance rate were determined in total and for each DDI pair.

#### End-user survey

An anonymous satisfaction e-survey was developed using Google Forms and sent via e-mail to all physicians (n = 1631). The survey was sent on February 9th 2021 and terminated on February 20th 2021. A reminder was sent after 1 week. Participation was voluntary and no reward was provided.

Initial questions were drafted by three clinical pharmacists (GVDS, LVDL, IS) and discussed with three independent clinical pharmacists (CQ, KW, EVL) and the chair of the Pharmacy & Therapeutics committee (MC), until full consensus was reached. Physicians were asked about general satisfaction, usefulness and relevance of the DDI module, as well as reasons for overriding very severe DDI alerts. The final questionnaire contained nine questions: two demographic questions, one 5-point Likert scale question, three multiple choice questions, two yes/no questions and one open-ended question. The survey questions are provided in Additional File 2.

### Data analysis

Quantitative data were analyzed using descriptive statistics. Answers on the open-ended survey question were analyzed using thematic analysis.

## Results

### Quantitative evaluation

During the 2-year study period, 110,400 patients were hospitalized in UZ Leuven, 232,948 patients were admitted to the day care hospital and 4,241,087 prescriptions were created.

A total of 38,409 very severe DDI alerts, divided over 64 DDI pairs, were generated. For 33,888 alerts (88.2%) an override was entered by the prescriber, resulting in an initial acceptance rate of 11.8%. For 23 DDI pairs, alerts were generated more than 100 times during the study period. The number of alerts and percentage of overrides for these 23 DDI pairs, are shown in Table 1. The complete table of the number of alerts and percentage of overrides for all 64 DDI pairs is provided in Additional File 3.

**Table 1:** Drug–drug interaction alerts and override rates

DDI pair	DDI alerts (n)	Prescribers’ overrides (%)
Factor Xa inhibitor + other anticoagulant	16,859	92.4
QTc prolonging agent + antiarrhythmic agent (flecainide, sotalol) (QTc)	6547	80.6
Antiarrhythmic agent (flecainide, amiodarone, sotalol) + antipsychotic (QTc)	3151	88.5
Dabigatran + other anticoagulant	2284	90.7
Antiarrhythmic agent (flecainide, amiodarone, sotalol) + tricyclic and related antidepressant (QTc)	1931	90.9
Statin (simvastatin, atorvastatin) + non-azithromycin macrolides	1287	86.6
Quetiapine + strong CYP3A4 inhibitor	933	93.2
Statin (simvastatin, atorvastatin) + azole antifungal agent	672	94.5
Statin (simvastatin, rosuvastatin) + cyclosporine	655	82.6
Opioid + MAO inhibitor	421	66.7
CYP3A4 substrate + CYP3A4 inducer	342	92.4
Valproic acid + carbapenem	280	88.9
*Saccharomyces boulardii* + glucocorticoid (high dose)	254	75.2
Vitamin K antagonist + acetylsalicylic acid (analgetic dose)	245	88.2
Droperidol, pimozide + macrolide (QTc)	228	43.4
Intravenous calcium + ceftriaxone	200	88.0
Antiarrhythmic agent (flecainide, amiodarone, sotalol, propafenone) + quinolone (QTc)	176	82.4
Serotonergic antidepressant + linezolid	163	91.4
Colchicine + CYP3A4 inhibitor (strong)	158	91.1
Colchicine + macrolide	154	92.9
Factor Xa inhibitor + azole antifungal agent	150	91.3
Live vaccines + glucocorticoid	112	70.5
Alcohol containing drugs + disulfiram	110	61.8

DDI, drug–drug interaction; QTc, QTc interval prolonging drug–drug interaction

Alerts for the co-prescription of a non-vitamin K oral anticoagulant (NOAC) and another type of anticoagulant (i.e. a low molecular weight heparin (LMWH) in 96% of cases) accounted for 19,143 of 38,409 (49.8%) very severe DDI alerts. The majority of these alerts (92.2%) was overridden by the prescriber. The override motivation mostly provided for this DDI was “no concurrent therapy”, pointing to the substitution of one drug by another without any overlap.

Alerts for the co-prescription of two QTc interval prolonging drugs accounted for 12,227 of 38,409 (31.8%) very severe DDI alerts. These alerts were overridden in 83.6% of cases. Motivations included that the patient did not show a prolonged QTc interval, that the QTc interval was being monitored, that one of the interacting drugs was only administered pro re nata, that the interacting drug combination was initiated outside the hospital setting or that one of the interacting drugs had already been discontinued upon receiving the alert.

A total of 14,320 override motivations were reviewed in the CMA. For 13,867 (96.8%) alerts, no action was undertaken by the clinical pharmacist, who judged the DDI as irrelevant and/or the override as justified. For 453 (3.2%) alerts, a recommendation was provided to the treating physician, of which 79.2% was accepted. Table 2 shows the number of pharmacist recommendations and their acceptance rate for the 23 most alerted DDI pairs. The complete table of the number of pharmacist recommendations and their acceptance rate for all 64 DDI pairs is provided in Additional File 4.

**Table 2:** Pharmacist recommendations and acceptance rates

DDI pair	CMA reviews (n)	Pharmacists’ recommendations (n (%))	Acceptance (%)
Factor Xa inhibitor + other anticoagulant	7216	80 (1.1)	100
QTc prolonging agent + antiarrhythmic agent (flecainide, sotalol) (QTc)	2283	72 (3.2)	80.4
Antiarrhythmic agent (flecainide, amiodarone, sotalol) + antipsychotic (QTc)	834	38 (4.6)	78.4
Dabigatran + other anticoagulant	943	8 (0.8)	100
Antiarrhythmic agent (flecainide, amiodarone, sotalol) + tricyclic and related antidepressant (QTc)	631	26 (4.1)	57.1
Statin (simvastatin, atorvastatin) + non-azithromycin macrolides	477	60 (12.6)	63.5
Quetiapine + CYP3A4 inhibitor	319	32 (10.0)	76.0
Statin (simvastatin, atorvastatin) + azole antifungal agent	209	17 (8.1)	71.4
Statin (simvastatin, rosuvastatin) + cyclosporine	199	10 (5.0)	66.7
Opioid + MAO inhibitor	92	5 (5.4)	100
CYP3A4 substrate + CYP3A4 inducer	107	19 (17.8)	73.3
Valproic acid + carbapenem	63	10 (15.9)	80.0
*Saccharomyces boulardii* + glucocorticoid (high dose)	69	2 (2.9)	0
Vitamin K antagonist + acetylsalicylic acid (analgetic dose)	78	2 (2.6)	100
Droperidol, pimozide + macrolide (QTc)	57	0 (0)	NA
Intravenous calcium + ceftriaxone	60	2 (3.3)	100
Antiarrhythmic agent (flecainide, amiodarone, sotalol, propafenone) + quinolone (QTc)	56	5 (8.9)	75.0
Serotonergic antidepressant + linezolid	40	6 (15.0)	20.0
Colchicine + CYP3A4 inhibitor (strong)	48	3 (6.3)	100
Colchicine + macrolide	74	2 (2.7)	100
Factor Xa inhibitor + azole antifungal agent	40	4 (10.0)	75.0
Live vaccine + glucocorticoid	26	0 (0)	NA
Alcohol containing drugs + disulfiram	30	0 (0)	NA

DDI, drug–drug interaction; CMA, Check of Medication Appropriateness; QTc, QTc interval prolonging drug–drug interaction

Among all override motivations, 8159 (57.0%) concerned the interaction between a NOAC and another anticoagulant. A recommendation was given to the treating physician for 88 (1.1%) alerts because co-prescription was indeed present. All of these recommendations (100%) were accepted.

Another 3959 of 14,320 (27.6%) motivations for overridden very severe DDI alerts concerned the interaction of two QTc prolonging drugs. In 146 (3.7%) cases, a recommendation was given to the treating physician because the QTc interval was indeed prolonged, or because the QTc interval was not documented under the combination of both drugs. The acceptance rate of these recommendations was 76.2%.

For 24 DDI pairs, the mean prescriber and pharmacist override rate was > 90% (Additional File 5). Amongst these were the interaction between a NOAC and another type of anticoagulant and the interaction between two QTc interval prolonging drugs.

### Results of the end-user survey

The satisfaction survey was completed by 175 physicians, corresponding to a response rate of 10.6%. Two surveys, completed by a dentist and an intensivist, were excluded from analysis because they reported to have no experience with the DDI CDSS system. Of the 173 physicians, 84 (48.6%) were medical staff members and 89 (51.4%) were physicians in training.

**Fig. 2:**
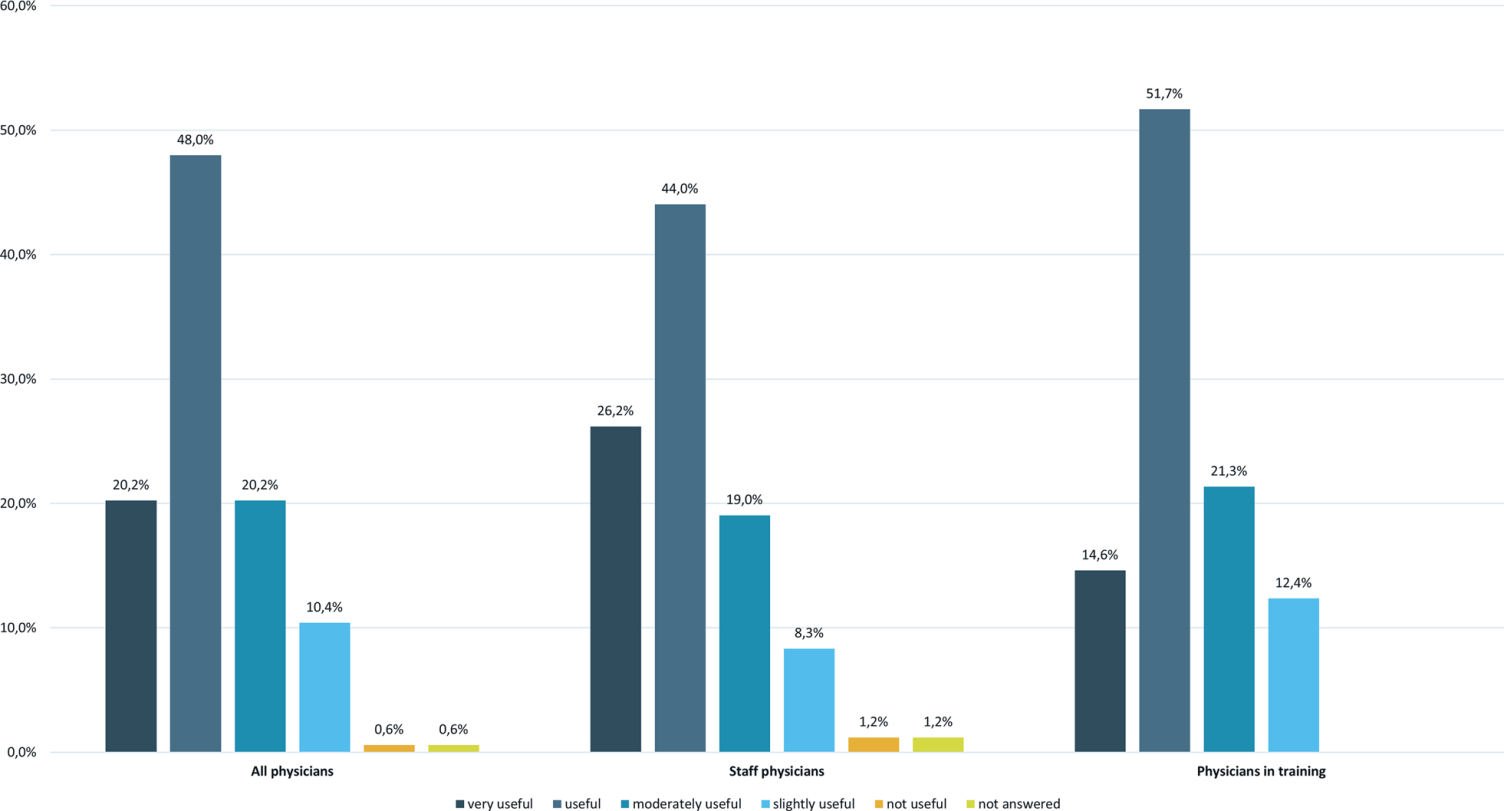
General experience of the drug–drug interaction module

The general experience of the DDI module was scored as moderately useful to very useful by 153 (88.4%) respondents (Fig. 2).

Eighty-four (48.6%) respondents reported to have changed a prescription based on a DDI alert in the past 3 months while 78 (45.1%) respondents did not change a prescription. Ten (5.8%) respondents did not remember whether they did.

**Fig. 3:**
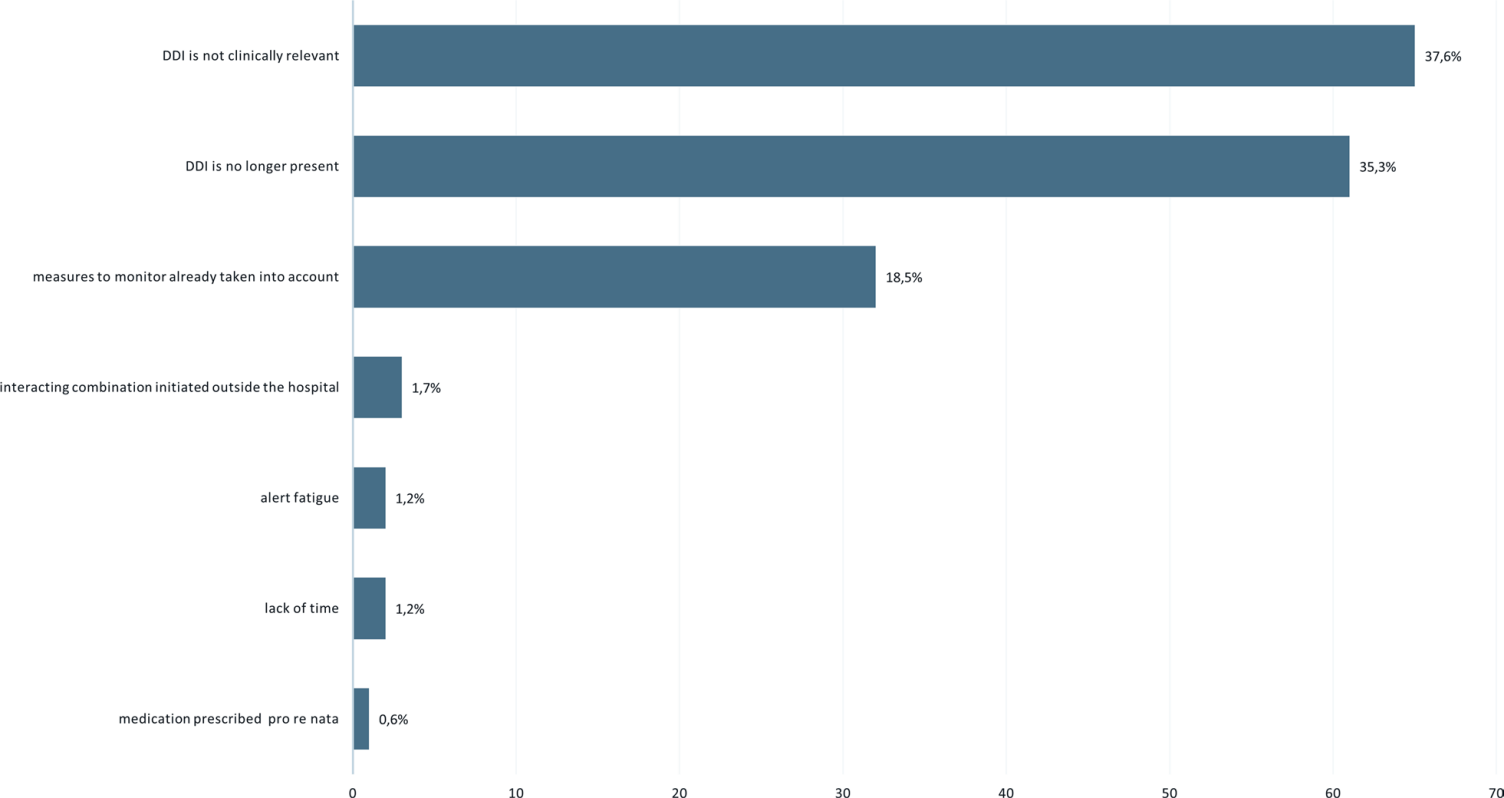
Main reasons for overruling a drug–drug interaction alert. DDI, drug–drug interaction

The main reasons for overriding a DDI alert were that (1) the DDI was considered to be not clinically relevant (n = 65, 37.6%) and (2) the DDI was no longer present (n = 61, 35.3%) (Fig. 3).

**Fig. 4:**
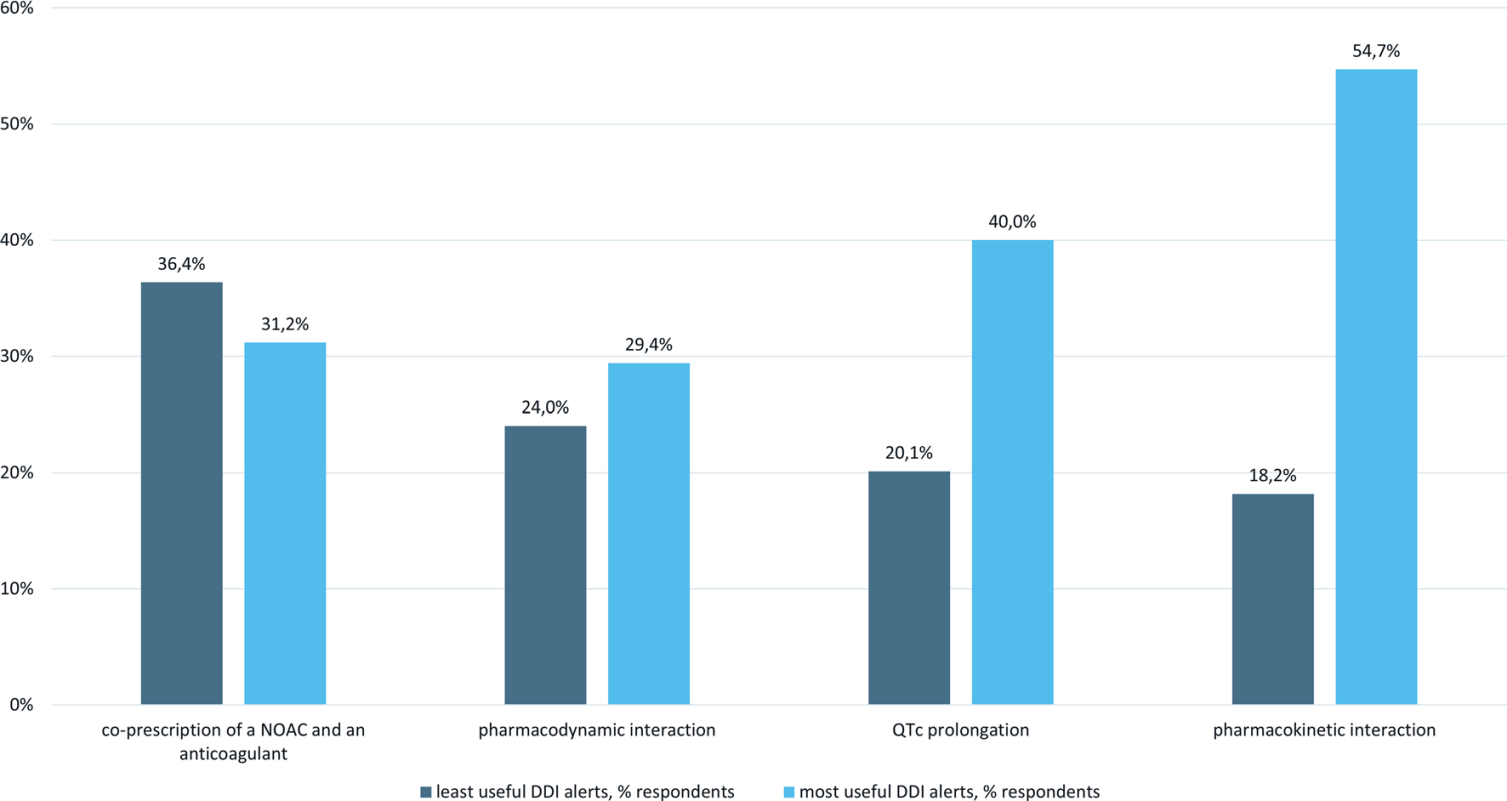
Type of drug–drug interaction alerts considered least useful and most useful. DDI, drug–drug interaction; NOAC, non-vitamin K oral anticoagulant

The question on the type of DDI alerts that was considered least useful was answered by 154 (89.0%) respondents with a total of 170 responses (Fig. 4). Co-prescription of a NOAC and an anticoagulant was considered the least useful DDI alert by 56 (36.4%) respondents. Seven (4.5%) respondents stated that all alerts were useful and another seven (4.5%) responded with another category of CDSS alerts (pregnancy and maximum dosing alerts), the latter being out of scope of the current evaluation.

In contrast, 170 (98.3%) respondents mentioned in 274 responses which DDIs were considered very useful (Fig. 4). A pharmacokinetic interaction was considered the most useful DDI alert by 93 (54.7%) respondents. The co-prescription of a NOAC and an anticoagulant was considered the most useful DDI alert by 53 (31.2%) respondents. Three (1.8%) respondents mentioned another category of CDSS alerts (pregnancy, maximum dosing alerts and kidney insufficiency).

Of all respondents that considered the co-prescription of a NOAC and an anticoagulant the least useful DDI alert, 44.6% were internists, 12.5% were surgeons, 12.5% were pediatricians and 10.7% were anesthesiologists. Of all respondents that considered the co-prescription of a NOAC and an anticoagulant the most useful DDI alert, 37.7% were internists, 30.2% were surgeons and 11.3% were anesthesiologists.

The detailed information/scientific evidence of a DDI alert was consulted in the past 3 months by 27 (15.6%) respondents, was not consulted by 131 (75.7%) respondents and 14 (8.1%) respondents did not remember.

The open-ended question for additional feedback resulted in 38 responses by 34 (19.7%) respondents. Responses were divided into 12 categories (Additional File 6). Respondents most often suggested the incorporation of a DDI-specific screening interval (n = 7, 20.6%), and the incorporation of patient-specific characteristics in the current DDI module (n = 7, 20.6%).

## Discussion

Our study showed that the current CDSS for very severe DDIs had a limited initial acceptance rate of 11.8%. The overrides were justified in most cases which was clearly illustrated by the very low pharmacist recommendation rate of 3.2%. The quantitative study showed a high override rate (mean > 90%) by both the prescriber and the reviewing pharmacist for two types of frequently generated DDI alerts. Alerts for the combination of two anticoagulants prescribed within the 14-day screening interval but without actual co-prescription were overridden because the interaction was not applicable, necessitating the use of DDI-specific screening intervals. Alerts for the combination of two QTc interval prolonging agents were overridden because the interaction was considered not clinically relevant (i.e. QTc interval not prolonged) or when measures to monitor the potential clinical consequence of the interaction were already put in place (i.e. a follow-up ECG), necessitating the inclusion of patient-specific characteristics (i.e. a recent QTc-value) into the DDI CDSS algorithms.

These results were supported by the results of the end-user survey, in which prescribers stated that reasons for overruling were that the DDI was considered not clinically relevant, was no longer applicable or measures to monitor were already taken into account. Surprisingly, the survey revealed that the overall satisfaction concerning the DDI CDSS was high.

**Fig. 5:**
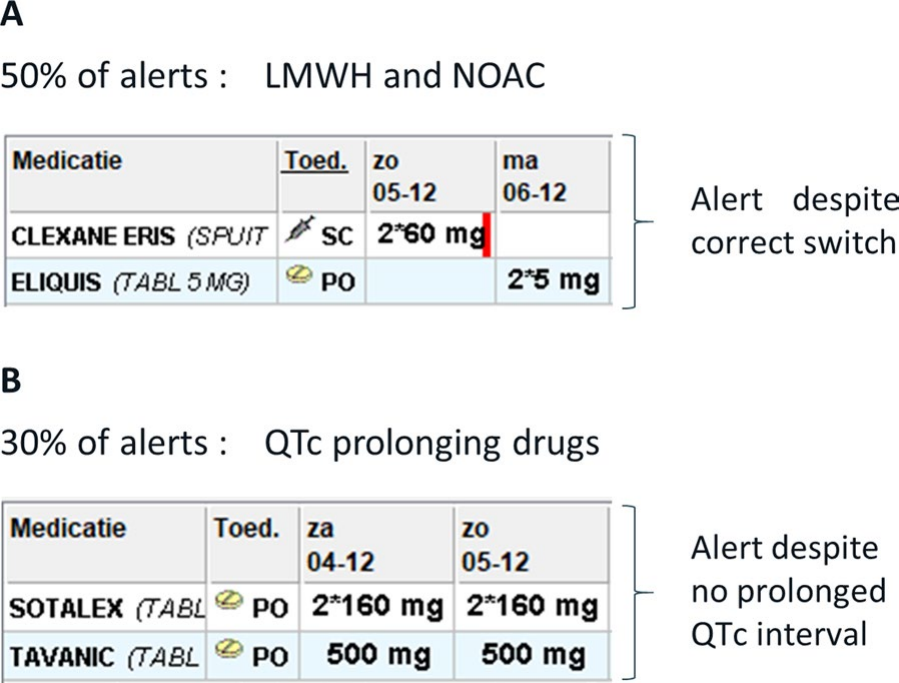
Main barriers of the current drug–drug interaction clinical decision support system. Panel **A**: a too broad screening interval leading to false positive alerts when switching correctly between anticoagulants. Panel **B**: lack of incorporation of patient-specific characteristics leading to false positive alerts when two QTc-prolonging drugs are prescribed for a patient whose QTc-interval is not prolonged

Hence, this analysis revealed a considerable alert burden and risk for alert fatigue mainly due to two major barriers of the current DDI CDSS system: a too broad screening interval of 14 days, generating an alert even when actual co-prescription is not present, and the lack of incorporation of patient-specific characteristics or measures undertaken to monitor the interaction e.g. generating an alert for QTc-prolongation even when the patient’s QTc-interval is normal (Fig. 5). Despite these barriers, the DDI CDDS was found useful by prescribers.

Approximately half of the very severe DDI alerts warned for an increased bleeding risk due to the apparent co-prescription of two anticoagulants. In 7.8% of cases, the alert was accepted and the prescription was canceled, whereas 92.2% of alerts were overridden. Of these prescribers’ overrides 98.9% were also overridden by the pharmacist. The main override motivation provided by prescribers was “no concurrent therapy”, indicating a switch between anticoagulants. Such switch is very common in the hospital setting, mostly as part of periprocedural bridging [24]. However, owing to the fixed screening interval of 14 days in the CPOE, false positive alerts are generated even when the switch of anticoagulants is executed correctly, i.e. when a NOAC and LMWH are prescribed at least 12 h apart (i.e. without actual co-prescription). However, in our study, in 88 (1.1%) cases actual co-prescription was present, with a high risk of major bleeding, even though the same override motivation (i.e. “no concurrent therapy”) was formulated. In our opinion, this strongly indicates the presence of alert fatigue, due to an overload of false positive alerts. Moreover, it underlines the added value of a secondary review of overridden DDI alerts by a specified trained person (e.g. a pharmacist) [25].

The co-prescription of two QTc interval prolonging drugs accounted for 31.8% of very severe DDI alerts of which 83.6% were overridden. Several override motivations were provided including that the patient did not show a prolonged QTc interval. Importantly, the QTc interval is not taken into account in the DDI module. Accordingly, an alert is shown even when a recent ECG shows no prolonged QTc interval. This lack of data integration compounds the already high burden of non-actionable alerts. After DDI review by the CMA pharmacist, a recommendation was given to the treating physician in 3.7% of the cases because of a prolonged QTc interval or the absence of a recent QTc measurement.

Questioning on the most and least useful DDI alerts in the survey yielded mixed results. Alerting for co-prescription of a NOAC and an anticoagulant was found to be most useful by 31.2% of respondents. Paradoxically, the same DDI pair was scored as least useful by 36.4% of respondents. Possible explanations are that that alert is only perceived to be useful when truly so and not useful when it concerns a false positive alert; and that usefulness depends on the type of medical specialist who receives the alert, along with knowledge on this specific drug combination [26]. The latter explanation is supported by our results in which 30.2% of respondents that considered a DDI alert for the co-prescription of a NOAC and an anticoagulant to be the most useful were surgeons.

The reported reasons for overriding a DDI alert aligned with the observed findings in our quantitative evaluation. In the open-ended question, the most common suggestions were to apply a DDI-specific screening interval and to incorporate patient-specific characteristics into the CDSS algorithms, which also supported the results found in the quantitative evaluation.

Surprisingly, the survey revealed that the overall satisfaction concerning the DDI CDSS was positive. The DDI CDSS was scored as moderately useful to very useful by 88.4% of respondents. This can be explained by two reasons. First, the added value of a DDI CDSS is recognized by prescribers despite its shortcomings. Secondly, alerts can be overridden by the physician upon prescribing but may trigger the physician to take specific actions afterwards and prior to CMA review, such as stopping one drug or ordering a follow-up ECG. These DDI alerts are then considered as overridden, although the DDI alert was relevant and led to a specific action. Our observed high physician override rates, and low pharmacist recommendation rates do not, by definition, imply that all of these alerts were irrelevant or false positive.

Our study results are in agreement with previous reports. High override rates of 56.3%-95.7% have already been described for DDI alerts [13, 21, 27–32]. Edrees et al. conducted a retrospective study of overridden high-priority DDI alerts and found an override rate of 87.3% for the highest severity DDI alerts [27]. More than half of the generated DDI alerts concerned the interaction between ondansetron and QTc prolonging agents and these were overridden in 96.5% of cases. Also Wong et al. [31] showed that most alerts (86.9%) were triggered by medication combinations that increase the risk of QTc prolongation.

Override appropriateness rate was higher in our study compared to the literature. Edrees et al. [27], Nanji et al. [29] and Wong et al. [31] found override appropriateness rates of 45.4%, 62% and 82%, respectively. In our study, only 3.2% of very severe DDI overrides resulted in an additional recommendation by the pharmacist, corresponding to an override appropriateness rate of 96.8%. Consequently, many overrides are considered justified. Likewise, an unacceptably high burden of irrelevant alerts is now present in our DDI CDSS system.

The strength of our study is that it comprises both a large-scale quantitative evaluation as well as an end-user satisfaction survey. The main results found in our quantitative evaluation were supported by the results of our survey. The quantitative evaluation has high internal validity and patient selection bias was low. An abundance of data was analyzed. Alert burden, initial acceptance and override rates were measured in total and also for each DDI pair separately. We do not only present data on alert burden and initial prescriber override/acceptance rates, but also on the secondary review of overridden DDI alerts by the pharmacist, the number of pharmacist recommendations and the secondary) override/acceptance rates of these recommendations. We present data on override appropriateness rates in a real-life setting (i.e. alerts overridden by both the prescriber and the pharmacist in the daily practice), unlike many other studies in which override appropriateness is evaluated retrospectively by medical record review by researchers. Furthermore, our study differs from numerous prior studies showing high override rates by pointing out the fundamental problems of the DDI CDSS that lead to a high number of false positive alerts.

Our study has the following limitations. First, only very severe DDIs were analyzed. However, very severe DDIs are the most burdensome as a free-text override motivation with password confirmation is required to continue the medication order. Besides, the two major barriers found in the analysis of very severe DDI alerts also apply for severe and other DDI alerts. Secondly, prescribers’ override motivations were not thoroughly evaluated in this study. Override motivations contain very useful information that can be used to optimize the DDI system. Analysis of these motivations was not possible due to the extent of our study and the obligation to override with free text. Thirdly, in the end-user survey study, a limited response rate of 10% was obtained, so self-selection bias cannot be excluded. The open-ended question was only answered by 34 prescribers. This limits the generalisability of these results.

Different barriers and improvement strategies of medication related CDSSs are described in literature. Similarly to the results of Mille et al. [30], the lack of DDI-specific screening intervals and lack of incorporation of context factors were identified, in both our quantitative evaluation and end-user survey, as the main barriers of our current DDI CDSS system. Recent studies already showed the potential of including patient-specific and context-specific characteristics into DDI CDSS algorithms on reducing the DDI alert burden [17, 18]. Chou et al. showed a reduction in DDI alerts by more than 50% [17]. Alerts were reduced by up to 93.5% in the study of Horn and Ueng [18]. Shah et al. compared two commercial CDSSs and showed a 94% reduction in alert burden, with much higher sensitivity and specificity [16]. A study by Muylle et al. recently showed a significant increase in alert acceptance, i.e. from 6.3 to 25.5%, by including DDI-specific screening intervals and contextual factors [15]. This is the only study, to our knowledge, investigating the effect of DDI-specific screening intervals. Specifically for QT-prolonging DDI alerts, Berger et al. [33] and Vandael et al. [34] recently developed models including several patient-specific risk factors to create smarter alerts concerning QTc prolongation.

Our future perspective is (i) to implement DDI-specific screening intervals for each DDI or DDI group taking into account several factors influencing the occurrence and/or duration of a DDI (e.g. the mechanism of the DDI, the half-live of the involved drugs and the sequence in which the interacting drugs are prescribed) and (ii) to include and take into account patient-specific characteristic into our CDSS system such as ECG and laboratory values in order to improve the alert burden and specificity of this system and to reduce alert fatigue.

## Conclusion

A broad screening interval and lack of incorporation of patient-specific characteristics were found to be the main barriers of the current DDI CDSS system, both leading to a high number of false positive alerts and risk for alert fatigue. Despite these barriers, the added value of the DDI CDSS was recognized by prescribers. Hence, efforts to further improve performance of DDI CDSSs are warranted.

## Supplementary Information

**Additional file 1: **Results of in-house re-categorization of DDI severity. 

**Additional file 2:** Survey questions, content and type of e-survey questions.

**Additional file 3: Table S1.** The complete table of the number of alerts and percentage of overrides for all 64 DDI pairs.

**Additional file 4: Table S2.** The complete table of the number of pharmacist recommendations and their acceptance rate for of all 64 DDI pairs.

**Additional file 5: Table S3.** DDI pairs for which the mean prescriber and pharmacist override rate was >90%.

**Additional file 6: Table S4.** Results of the open-ended survey question.

## Data Availability

All data generated or analyzed in this study are included in the published article and/or in the Additional Files.

## Abbreviations

BMUC: Belgian Meaningful Use Criteria
CDSS: Clinical decision support system
CMA: Check of Medication Appropriateness
CPOE: Computerized physician order entry
DDI: Drug–drug interaction
KWS: Klinisch Werkstation
LMWH: Low molecular weight heparin
NOAC: Non-vitamin K oral anticoagulant
UZ Leuven: University Hospitals Leuven
