# Supplementary material for: Overall performance of a drug–drug interaction clinical decision support system: quantitative evaluation and end-user survey
Source: BMC Med Inform Decis Mak. 2022 Feb 22;22:48. doi: 10.1186/s12911-022-01783-z (PMC8864797; doi:10.1186/s12911-022-01783-z)
Supplement: Supplementary file 1 — Additional file 1: Results of in-house re-categorization of DDI severity. [file 12911_2022_1783_MOESM1_ESM.docx]

**ADDITIONAL FILE 1**

**Results of in-house re-categorization of DDI severity**

Our drug-drug interaction clinical decision support system makes use of the DelphiCare® database. In this database, DDIs were categorized in six groups based on severity till October 2013. From October 2013, DDIs were categorized in eight risk groups.

| Severity level classification DelphiCare® database till October 2013 | |
| --- | --- |
| 1 | Contraindicated |
| 2 | Contraindicated by precaution |
| 3 | Patient monitoring or adaptation of drug regimen required |
| 4 | Patient monitoring or adaption of drug regimen required in specific cases only |
| 5 | Proceed with caution |
| 6 | No measures needed |

| Severity level classification DelphiCare® database from October 2013 | |
| --- | --- |
| 1 | Contraindicated |
| 2 | Contraindicated in specific cases |
| 3 | Contraindicated by precaution |
| 4 | Concurrent use not recommended |
| 5 | Patient monitoring or adaptation of drug regimen required |
| 6 | Patient monitoring or adaption of drug regimen required in specific cases only |
| 7 | Proceed with caution |
| 8 | No measures needed |

In our home-grown hospital information system, only three severity categories are used to classify DDIs (i.e. “very severe”, “severe” and “other” DDIs).

The expert working group consists of physicians and hospital pharmacists and gathers on a regular basis since 2009. During these meetings, the drug-drug interactions with a severity category of 1, 2 or 3 based on DelphiCare® are reviewed based on the available literature and are then allocated to the “very severe”, “severe” or “other” DDI group. Interactions with a lower severity category according to the DelphiCare® database (i.e. category 4-6 and 4-8 before and after October 2013) are not routinely reviewed but are automatically assigned to the “other” DDI group. These DDIs can however be allocated to a higher severity group at the request of physicians or hospital pharmacists.

Criteria used by the expert working group are the severity of the potential consequences of the drug-drug interaction and the available evidence.

Today we have 2066 drug-drug interaction groups in our drug-drug interaction clinical decision support system:

| **DelphiCare® severity level** | **Customized in-house severity level** |
| --- | --- |
| 81 DDIs with severity level 1 | 56 “very severe” |
|  | 15 “severe” |
|  | 10 “other” |
| 17 DDIs with severity level 2 | 4 “very severe” |
|  | 7 “severe” |
|  | 6 “other” |
| 188 DDIs with severity level 3 | 46 “very severe” |
|  | 74 “severe” |
|  | 68 “other” |
| 363 DDIs with severity level 4 | 20 “very severe” |
|  | 54 “severe” |
|  | 289 “other” |
| 650 DDIs with severity level 5 | 5 “very severe” |
|  | 139 “severe” |
|  | 506 “other” |
| 65 DDIs with severity level 6 | 4 “severe” |
|  | 61 “other” |
| 674 DDIs with severity level 7 | 1 “very severe” |
|  | 15 “severe” |
|  | 658 “other” |
| 28 DDIs with severity level 8 | 28 “other” |

All DDIs with a DelphiCare® severity level of 1, 2 or 3 (i.e. 286 DDIs) have been reviewed by the expert working group. Some DDIs with a lower severity level (today) have also been reviewed by the expert working group, (i) because they had higher DelphiCare® severity level assigned before October 2013 or (ii) because the DDI was reviewed at the request of a physician or hospital pharmacist.
