# Supplementary material for: Overall performance of a drug–drug interaction clinical decision support system: quantitative evaluation and end-user survey
Source: BMC Med Inform Decis Mak. 2022 Feb 22;22:48. doi: 10.1186/s12911-022-01783-z (PMC8864797; doi:10.1186/s12911-022-01783-z)
Supplement: Supplementary file 2 — Additional file 2: Survey questions, content and type of e-survey questions. [file 12911_2022_1783_MOESM2_ESM.docx]

**ADDITIONAL FILE 2**

**Survey Questions**

Demographic questions

1) Are you a medical staff member or a physician in training?

2) Which is your medical specialty? (multiple choice, one answer allowed)

Questions on the drug-drug interaction clinical decision support system

1) How do you experience the DDI feature? (5-point Likert scale)

2) Did you change a prescription due to a DDI alert in the last 3 months? (yes/no)

3) What is the most important reason to override a DDI alert? (multiple choice, one answer allowed)

4) Which DDI alerts do you find the least useful? (multiple choice, multiple answers allowed)

5) Which DDI alerts do you find the most useful? (multiple choice, multiple answers allowed)

6) Did you proceed to the detail/scientific evidence of a DDI alert in the past 3 months? (yes/no)

7) Do you have any remarks or suggestions? (open-ended)

For the multiple choice questions an option for providing another answer was provided.
