# Supplementary material for: Overall performance of a drug–drug interaction clinical decision support system: quantitative evaluation and end-user survey
Source: BMC Med Inform Decis Mak. 2022 Feb 22;22:48. doi: 10.1186/s12911-022-01783-z (PMC8864797; doi:10.1186/s12911-022-01783-z)
Supplement: Supplementary file 3 — Additional file 3: Table S1. The complete table of the number of alerts and percentage of overrides for all 64 DDI pairs. [file 12911_2022_1783_MOESM3_ESM.docx]

**ADDITIONAL FILE 3**

**Table S1**

| **Table S1.** Drug-drug interaction alerts and override rates | | |
| --- | --- | --- |
| **DDI pair** | **DDI alerts (n)** | **Prescribers’ overrides (%)** |
| Factor Xa inhibitor + other anticoagulant | 16859 | 92.4 |
| QTc prolonging agent + antiarrhythmic agent (flecainide, sotalol) (QTc) | 6547 | 80.6 |
| Antiarrhythmic agent (flecainide, amiodarone, sotalol) + antipsychotic (QTc) | 3151 | 88.5 |
| Dabigatran + other anticoagulant | 2284 | 90.7 |
| Antiarrhythmic agent (flecainide, amiodarone, sotalol) + tricyclic and related antidepressant (QTc) | 1931 | 90.9 |
| Statin (simvastatin, atorvastatin) + non-azithromycin macrolides | 1287 | 86.6 |
| Quetiapine + CYP3A4 inhibitor | 933 | 93.2 |
| Statin (simvastatin, atorvastatin) + azole antifungal agent | 672 | 94.5 |
| Statin (simvastatin, rosuvastatin) + cyclosporine | 655 | 82.6 |
| Opioid + MAO inhibitor | 421 | 66.7 |
| CYP3A4 substrate + CYP3A4 inducer | 342 | 92.4 |
| Valproic acid + carbapenem | 280 | 88.9 |
| *Saccharomyces boulardii* + glucocorticoid (high dose) | 254 | 75.2 |
| Vitamin K antagonist + acetylsalicylic acid (analgetic dose) | 245 | 88.2 |
| Droperidol, pimozide + macrolide (QTc) | 228 | 43.4 |
| Intravenous calcium + ceftriaxone | 200 | 88.0 |
| Antiarrhythmic agent (flecainide, amiodarone, sotalol, propafenone) + quinolone (QTc) | 176 | 82.4 |
| Serotonergic antidepressant + linezolid | 163 | 91.4 |
| Colchicine + CYP3A4 inhibitor (strong) | 158 | 91.1 |
| Colchicine + macrolide | 154 | 92.9 |
| Factor Xa inhibitor + azole antifungal agent | 150 | 91.3 |
| Live vaccine + glucocorticoid | 112 | 70.5 |
| Alcohol containing drugs + disulfiram | 110 | 61.8 |
| Digitalis + intravenous calcium | 87 | 69.0 |
| Phosphodiesterase type 5 inhibitor + CYP3A4 inhibitor | 85 | 69.4 |
| Apixaban + inducer of CYP3A4 and P-glycoprotein (strong) | 84 | 88.1 |
| Antiarrhythmic agent + H1 antagonist (QTc) | 83 | 89.2 |
| Purine antagonist + xanthine oxidase inhibitor | 74 | 90.5 |
| Edoxaban + P-glycoprotein inducer | 74 | 91.9 |
| Dabigatran + P-glycoprotein inducer | 58 | 94.8 |
| *Saccharomyces boulardii* + immunosuppressant | 48 | 64.6 |
| Nitrate + phosphodiesterase type 5 inhibitor | 47 | 70.2 |
| Azole antifungal agent + rifampicin | 40 | 67.5 |
| Rilpivirin + proton pump inhibitor | 40 | 77.5 |
| QTc prolonging agent + fluconazole (QTc) | 40 | 60.0 |
| QTc prolonging agent + amiodaron, dronedarone (QTc) | 31 | 80.6 |
| Levodopa + MAO inhibitor (unselective and MAO-A selective) | 25 | 100 |
| Live vaccin + immunosuppressant | 25 | 0 |
| Doravirine, rilpivirine + CYP3A4 inducer (strong) | 24 | 66.7 |
| terlipressine + QTc prolonging agent (QTc) | 21 | 81.0 |
| Dabigatran + P-glycoprotein inhibitor (strong) | 20 | 95.0 |
| Pimozide + selective serotonin reuptake inhibitor (QTc) | 19 | 89.5 |
| Ivabradine + CYP3A4 inhibitor (strong) | 16 | 78.5 |
| Aromatase inhibitor + Tamoxifen | 15 | 80.0 |
| Clozapine + chemotherapy | 15 | 53.3 |
| Ticagrelor + CYP3A4 inhibitor (strong) | 14 | 100 |
| Voriconazol + phenytoin | 13 | 100 |
| Bupropion + MAO inhibitor | 12 | 91.7 |
| CYP3A4 substrate + cobicistat | 12 | 83.3 |
| Rifabutine + HIV protease inhibitor | 11 | 100 |
| Atovaquon + Rifamycin | 11 | 100 |
| Drugs with oxidative metabolism + ritonavir | 10 | 20.0 |
| Retinoid + tetracyclines | 8 | 100 |
| Sympathomimetic + MAO inhibitor (unselective) | 7 | 100 |
| Isavuconazol + CYP3A4 inducer | 5 | 100 |
| Serotonergic antidepressant + MAO inhibitor (irreversible) | 4 | 75.0 |
| Tizanidine + strong CYP1A2 inhibitor | 4 | 25.0 |
| Ergot alkoids + triptan | 3 | 100 |
| Antiarrhythmic agent + HIV-proteaseremmers | 3 | 100 |
| Flecainide + antiarrhythmic agent (class I) | 3 | 100 |
| Voriconazol + Rifampicin | 2 | 100 |
| Methylphenidate + proton pump inhibitor or antacid | 2 | 100 |
| Serotonergic antidepressant + moclobemide | 1 | 100 |
| Plasminogen activator + defibrotide | 1 | 100 |
| **Total** | **38409** | **88.2** |
| DDI, drug-drug interaction; QTc, QTc interval prolonging drug-drug interaction | | |
