# Supplementary material for: Overall performance of a drug–drug interaction clinical decision support system: quantitative evaluation and end-user survey
Source: BMC Med Inform Decis Mak. 2022 Feb 22;22:48. doi: 10.1186/s12911-022-01783-z (PMC8864797; doi:10.1186/s12911-022-01783-z)
Supplement: Supplementary file 4 — Additional file 4: Table S2. The complete table of the number of pharmacist recommendations and their acceptance rate for of all 64 DDI pairs. [file 12911_2022_1783_MOESM4_ESM.docx]

**ADDITIONAL FILE 4**

**Table S2**

| **Table S2.** Pharmacist recommendations and acceptance rates | | | |
| --- | --- | --- | --- |
| **DDI interaction pair** | **CMA reviews (n)** | **Pharmacists’ recommendations**  **(n (%))** | **Acceptance (%)** |
| Factor Xa inhibitor + other anticoagulant | 7216 | 80 (1.1) | 100 |
| QTc prolonging agent + antiarrhythmic agent (flecainide, sotalol) (QTc) | 2283 | 72 (3.2) | 80.4 |
| Antiarrhythmic agent (flecainide, amiodarone, sotalol) + antipsychotic (QTc) | 834 | 38 (4.6) | 78.4 |
| Dabigatran + other anticoagulant | 943 | 8 (0.8) | 100 |
| Antiarrhythmic agent (flecainide, amiodarone, sotalol) + tricyclic and related antidepressant (QTc) | 631 | 26 (4.1) | 57.1 |
| Statin (simvastatin, atorvastatin) + non-azithromycin macrolides | 477 | 60 (12.6) | 63.5 |
| Quetiapine + CYP3A4 inhibitor | 319 | 32 (10.0) | 76.0 |
| Statin (simvastatin, atorvastatin) + azole antifungal agent | 209 | 17 (8.1) | 71.4 |
| Statin (simvastatin, rosuvastatin) + cyclosporine | 199 | 10 (5.0) | 66.7 |
| Opioid + MAO inhibitor | 92 | 5 (5.4) | 100 |
| CYP3A4 substrate + CYP3A4 inducer | 107 | 19 (17.8) | 73.3 |
| Valproic acid + carbapenem | 63 | 10 (15.9) | 80.0 |
| *Saccharomyces boulardii* + glucocorticoid (high dose) | 69 | 2 (2.9) | 0 |
| Vitamin K antagonist + acetylsalicylic acid (analgetic dose) | 78 | 2 (2.6) | 100 |
| Droperidol, pimozide + macrolide (QTc) | 57 | 0 (0) | NA |
| Intravenous calcium + ceftriaxone | 60 | 2 (3.3) | 100 |
| Antiarrhythmic agent (flecainide, amiodarone, sotalol, propafenone) + quinolone (QTc) | 56 | 5 (8.9) | 75.0 |
| Serotonergic antidepressant + linezolid | 40 | 6 (15.0) | 20.0 |
| Colchicine + CYP3A4 inhibitor (strong) | 48 | 3 (6.3) | 100 |
| Colchicine + macrolide | 74 | 2 (2.7) | 100 |
| Factor Xa inhibitor + azole antifungal agent | 40 | 4 (10.0) | 75.0 |
| Live vaccin + glucocorticoid | 26 | 0 (0) | NA |
| Alcohol containing drugs + disulfiram | 30 | 0 (0) | NA |
| Digitalis + intravenous calcium | 23 | 1 (4.3) | 100 |
| Phosphodiesterase type 5 inhibitor + CYP3A4 inhibitor | 12 | 3 (25.0) | 50.0 |
| Apixaban + inducer of CYP3A4 and P-glycoprotein (strong) | 19 | 11 (57.9) | 77.8 |
| Antiarrhythmic agent + H1 antagonist (QTc) | 64 | 1 (1.6) | 100 |
| Purine antagonist + xanthine oxidase inhibitor | 42 | 4 (9.5) | 100 |
| Edoxaban + P-glycoprotein inducer | 19 | 1 (5.3) | 100 |
| Dabigatran + P-glycoprotein inducer | 24 | 5 (20.8) | 60.0 |
| *Saccharomyces boulardii* + immunosuppressant | 8 | 3 (37.5) | 100 |
| Nitrate + phosphodiesterase type 5 inhibitor | 17 | 0 (0) | NA |
| Azole antifungal agent + rifampicin | 11 | 1 (9.1) | 0 |
| Rilpivirin + proton pump inhibitor | 16 | 4 (25.0) | 100 |
| QTc prolonging agent + fluconazole (QTc) | 6 | 1 (16.7) | 100 |
| QTc prolonging agent + amiodaron, dronedarone (QTc) | 17 | 3 (17.6) | 100 |
| Levodopa + MAO inhibitor (unselective and MAO-A selective) | 9 | 2 (22.2) | 100 |
| Live vaccin + immunosuppressant | 0 | NA | NA |
| Doravirine, rilpivirine + CYP3A4 inducer (strong) | 5 | 0 (0) | NA |
| terlipressine + QTc prolonging agent (QTc) | 4 | 0 (0) | NA |
| Dabigatran + P-glycoprotein inhibitor (strong) | 7 | 2 (28.6) | NA |
| Pimozide + selective serotonin reuptake inhibitor (QTc) | 7 | 0 (0) | NA |
| Ivabradine + CYP3A4 inhibitor (strong) | 4 | 0 (0) | NA |
| Aromatase inhibitor + Tamoxifen | 9 | 0 (0) | NA |
| Clozapine + chemotherapy | 2 | 0 (0) | NA |
| Ticagrelor + CYP3A4 inhibitor (strong) | 6 | 0 (0) | NA |
| Voriconazol + phenytoin | 4 | 1 (25.0) | 100 |
| Bupropion + MAO inhibitor | 4 | 1 (25.0) | NA |
| CYP3A4 substrate + cobicistat | 4 | 0 (0) | NA |
| Rifabutine + HIV protease inhibitor | 3 | 1 (33.3) | NA |
| Atovaquon + Rifamycin | 5 | 4 (80.0) | NA |
| Drugs with oxidative metabolism + ritonavir | 2 | 0 (0) | NA |
| Retinoid + tetracyclines | 4 | 0 (0) | NA |
| Sympathomimetic + MAO inhibitor (unselective) | 1 | 0 (0) | NA |
| Isavuconazol + CYP3A4 inducer | 2 | 0 (0) | NA |
| Serotonergic antidepressant + MAO inhibitor (irreversible) | 2 | 0 (0) | NA |
| Tizanidine + strong CYP1A2 inhibitor | 1 | 0 (0) | NA |
| Ergot alkoids + triptan | 0 | NA | NA |
| Antiarrhythmic agent + HIV-proteaseremmers | 2 | 0 (0) | NA |
| Flecainide + antiarrhythmic agent (class I) | 2 | 0 (0) | NA |
| Voriconazol + Rifampicin | 1 | 0 (0) | NA |
| Methylphenidate + proton pump inhibitor or antacid | 1 | 1 (100) | 100 |
| Serotonergic antidepressant + moclobemide | 0 | NA | NA |
| Plasminogen activator + defibrotide | 0 | NA | NA |
| **Total** | **14320** | **453 (3.2)** | **79.2** |
| DDI, drug-drug interaction; CMA, Check of Medication Appropriateness; QTc, QTc interval prolonging drug-drug interaction | | | |
