# Supplementary material for: Overall performance of a drug–drug interaction clinical decision support system: quantitative evaluation and end-user survey
Source: BMC Med Inform Decis Mak. 2022 Feb 22;22:48. doi: 10.1186/s12911-022-01783-z (PMC8864797; doi:10.1186/s12911-022-01783-z)
Supplement: Supplementary file 5 — Additional file 5: Table S3. DDI pairs for which the mean prescriber and pharmacist override rate was >90%. [file 12911_2022_1783_MOESM5_ESM.docx]

**ADDITIONAL FILE 5**

**Table S3**

| **Table S3.** Drug-drug interaction alerts with high override rates of both prescriber and pharmacist | | | | | |
| --- | --- | --- | --- | --- | --- |
| **DDI pair** | **DDI alerts (n)** | **Prescribers’ overrides (%)** | **CMA reviews (n)** | **Pharmacists’ overrides (%)** | **Mean override rate (%)** |
| Factor Xa inhibitor + other anticoagulant | 16859 | 92.4 | 7216 | 98.9 | 95.7 |
| Antiarrhythmic agent (flecainide, amiodarone, sotalol) + antipsychotic (QTc) | 3151 | 88.5 | 834 | 95.4 | 92.0 |
| Dabigatran + other anticoagulant | 2284 | 90.7 | 943 | 99.2 | 94.9 |
| Antiarrhythmic agent + tricyclic and related antidepressant (QTc) | 1931 | 90.9 | 631 | 95.9 | 93.4 |
| Quetiapine + CYP3A4 inhibitor | 933 | 93.2 | 319 | 90.0 | 91.6 |
| Statin (simvastatin, atorvastatin) + azole antifungal agent | 672 | 94.5 | 209 | 91.9 | 93.2 |
| Vitamin K antagonist + acetylsalicylic acid (high dose) | 245 | 88.2 | 78 | 97.4 | 92.8 |
| Intravenous calcium + ceftriaxone | 200 | 88.0 | 60 | 96.7 | 92.4 |
| Colchicine + CYP3A4 inhibitor (strong) | 158 | 91.1 | 48 | 93.7 | 92.4 |
| Colchicine + macrolide | 154 | 92.9 | 74 | 97.3 | 95.1 |
| Factor Xa inhibitor + azole antifungal agent | 150 | 91.3 | 40 | 90 | 90.7 |
| Antiarrhythmic agent + H1 antagonist (QTc) | 83 | 89.2 | 64 | 98.4 | 93.8 |
| Edoxaban + P-glycoprotein inducer | 74 | 91.9 | 19 | 94.7 | 93.3 |
| Purine antagonist + xanthine oxidase inhibitor | 74 | 90.5 | 42 | 90.5 | 90.5 |
| Terlipressine + QTc prolonging agent (QTc) | 21 | 81.0 | 4 | 100 | 90.5 |
| Pimozide + selective serotonin reuptake inhibitor (QTc) | 19 | 89.5 | 7 | 100 | 94.8 |
| Ticagrelor + CYP3A4 inhibitor (strong) | 14 | 100 | 6 | 100 | 100 |
| CYP3A4 substrate + cobicistat | 12 | 83.3 | 4 | 100 | 91.7 |
| Retinoid + tetracyclines | 8 | 100 | 4 | 100 | 100 |
| Sympathicomimetic + MAO inhibitor (unselective) | 7 | 100 | 1 | 100 | 100 |
| Isavuconazol + CYP3A4 inducer | 5 | 100 | 2 | 100 | 100 |
| Antiarrhythmic agent + HIV-proteaseremmers | 3 | 100 | 2 | 100 | 100 |
| Flecainide + antiarrhythmic agent (class I) | 3 | 100 | 2 | 100 | 100 |
| Voriconazol + rifampicin | 2 | 100 | 1 | 100 | 100 |
| DDI, drug-drug interaction; CMA, Check of Medication Appropriateness; QTc, QTc interval prolonging drug-drug interaction | | | | | |
