# Supplementary material for: Overall performance of a drug–drug interaction clinical decision support system: quantitative evaluation and end-user survey
Source: BMC Med Inform Decis Mak. 2022 Feb 22;22:48. doi: 10.1186/s12911-022-01783-z (PMC8864797; doi:10.1186/s12911-022-01783-z)
Supplement: Supplementary file 6 — Additional file 6: Table S4. Results of the open-ended survey question. [file 12911_2022_1783_MOESM6_ESM.docx]

**ADDITIONAL FILE 6**

**Table S4**

| **Table S4.** Open-ended survey question: do you have any remarks or suggestions about de drug-drug interaction module | |
| --- | --- |
| **Category** | **number of responses** |
| Suggestions | |
| DDI-specific screening interval | 7 |
| inclusion of patient-specific characteristics | 7 |
| easier manner of overruling | 3 |
| more clear design of alerts | 3 |
| development new CDSS feature | 1 |
| expand front-office clinical pharmacy services | 1 |
| more specific alerts | 1 |
| Remarks | |
| missing of specific DDIs | 2 |
| DDI present in readily available prescription schemes | 2 |
| Feedback | |
| positive feedback | 6 |
| negative feedback | 1 |
| Not applicable | |
| answer concerning other CDSS module | 4 |
| DDI, drug-drug interaction; CDSS, clinical decision support system | |
